# Supplementary material for: Development and validation of a prognostic and predictive 32-gene signature for gastric cancer
Source: Nat Commun. 2022 Feb 9;13:774. doi: 10.1038/s41467-022-28437-y (PMC8828873; doi:10.1038/s41467-022-28437-y)
Supplement: Supplementary file 1 — Supplementary Information [file 41467_2022_28437_MOESM1_ESM.pdf]

Supplementary Figure 1

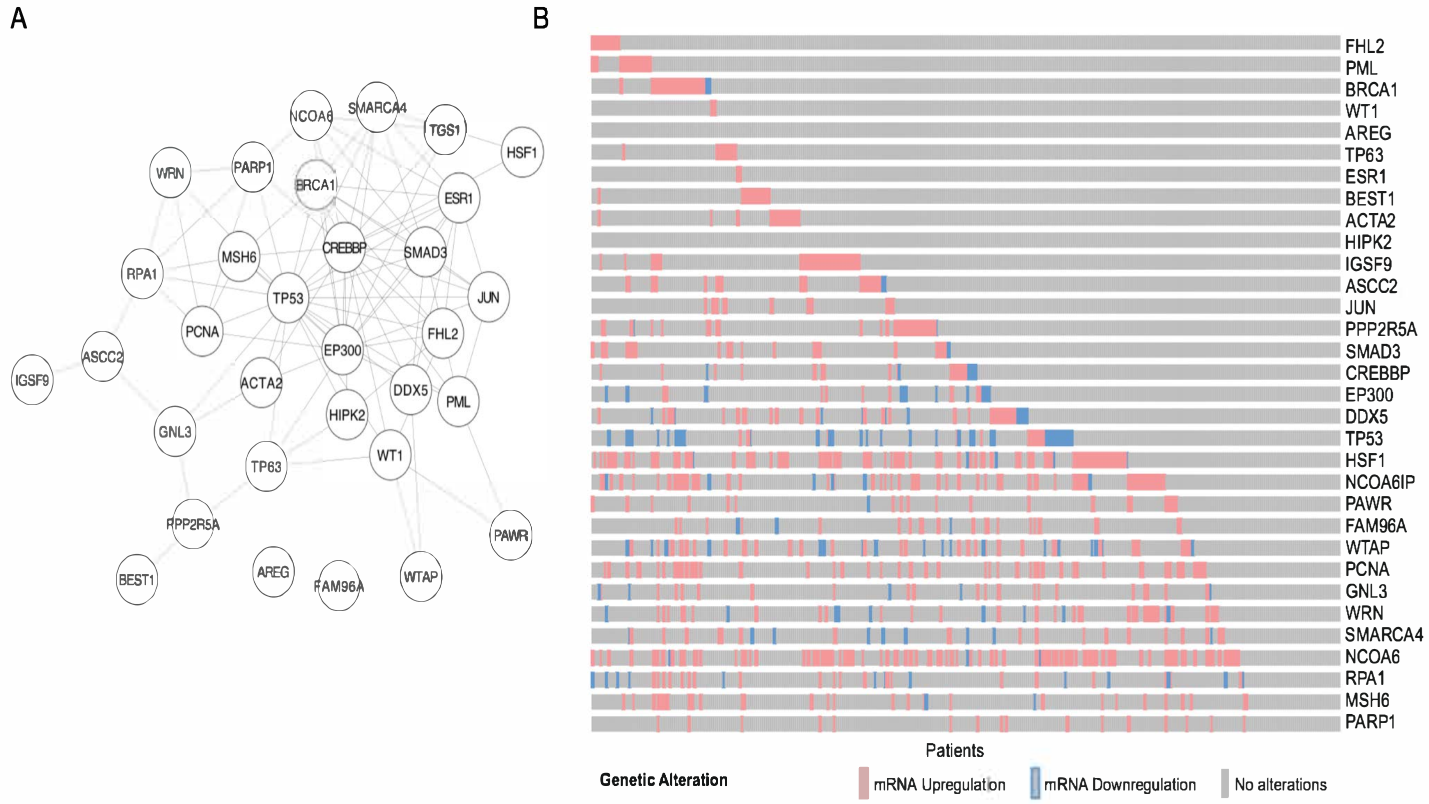


**Supplementary Figure 1.**
(A) The 32 genes that comprised the top three altered gastric cancer specific pathways.
(B) mRNA expression status for 32 genes in the TCGA adenocarcinoma cohort. Each row represents a member of the 32 gene signature, and each column represents a patient sample in the TCGA gastric adenocarcinoma cohort.

**Supplementary Figure 2**

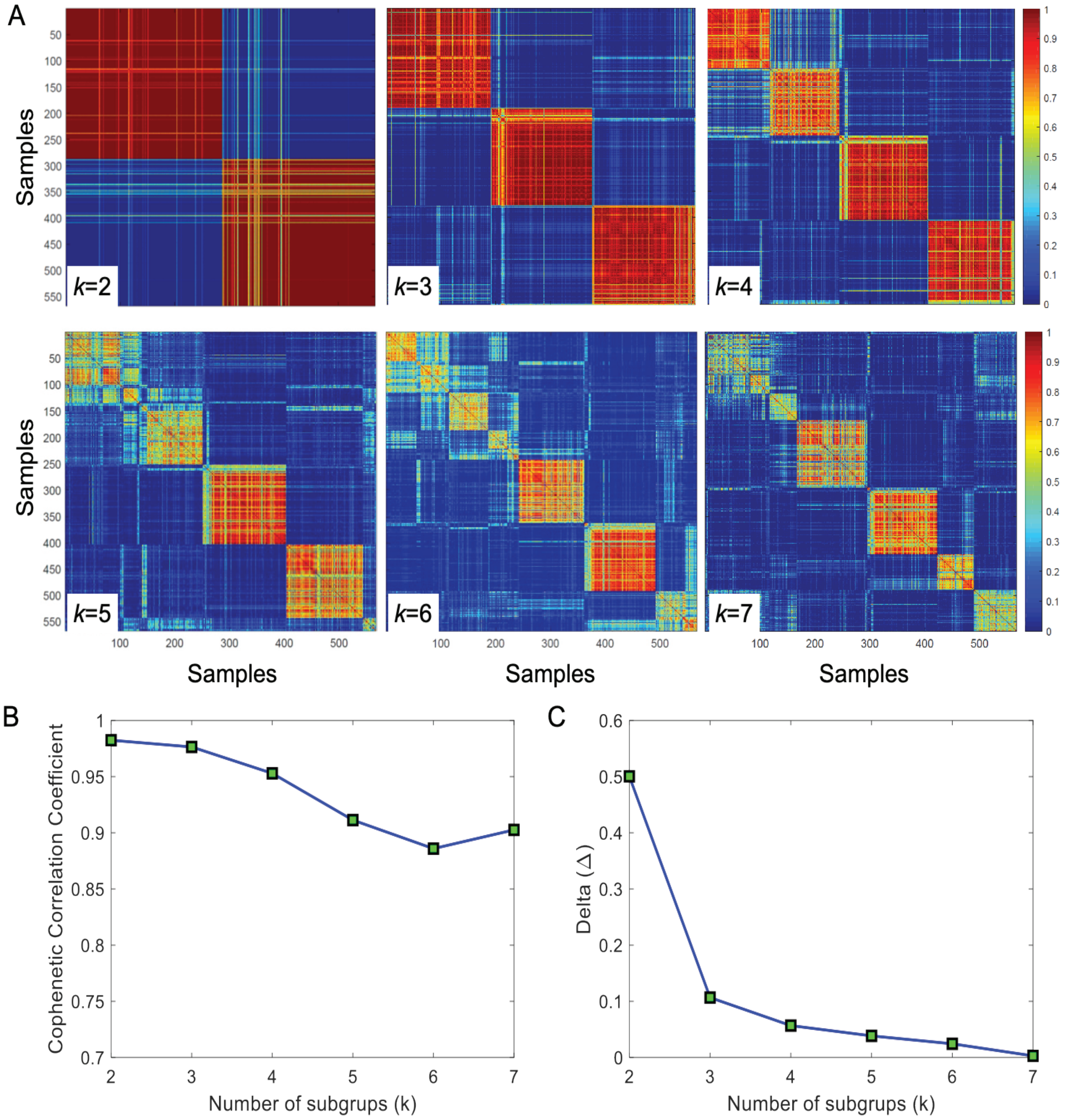


**Supplementary Figure 2.**
Unsupervised consensus clustering of the Yonsei cohort.
(A) Consensus matrix heat maps based on the number of clusters (from k=2 to 7).
(B) Cophenetic correlation coefficient under corresponding k values.
(C) The relative change in area under the cumulative distribution function (CDF) curves (e.g., delta value) when the cluster number varies from k=2 to 7.

**Supplementary Figure 3**

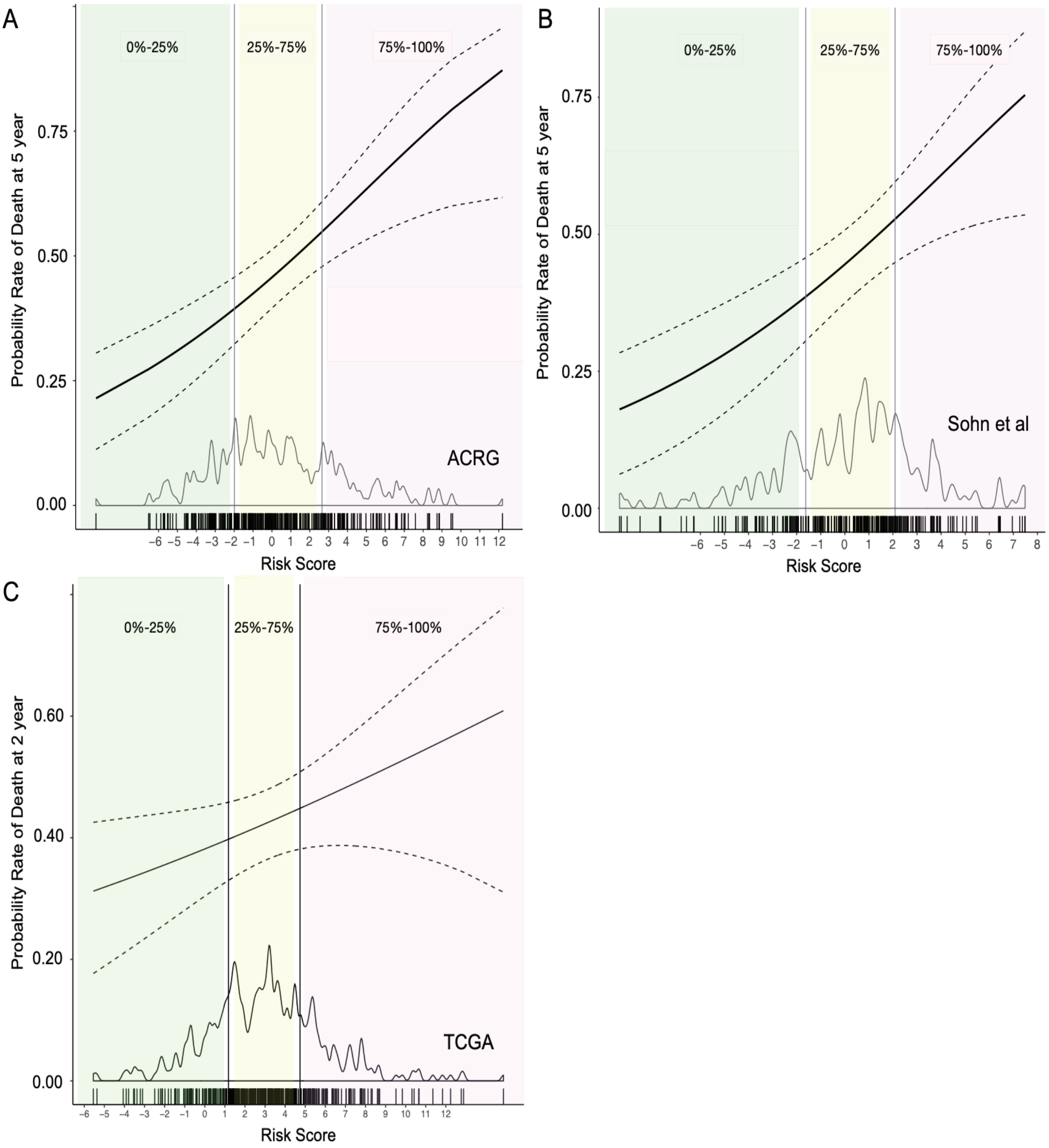


**Supplementary Figure 3.**
Rate of death at five years as a function of the risk score for the (A) ACRG, (B) Sohn et al, and (C) TCGA STAD cohort. The dashed curves indicate the 95% confidence interval. The rug plot on top of the x-axis shows the risk score for individual patients. The green region represents patients with scores below the 25th percentile, the yellow area includes patients with scores from the 25th to 75th percentile, and the purple region includes patients with scores above the 75th percentile.

**Supplementary Table 1. Cancer types analyzed by NTriPath**

Acute Myeloid Leukemia (LAML)
Bladder Urothelial Carcinoma (BLCA)
Brain Lower Grade Glioma (LGG),
Breast Invasive Carcinoma (BRCA)
Cervical Squamous Cell Carcinoma and Endocervical Adenocarcinoma (CESC)
Colon Adenocarcinoma (COAD)
Glioblastoma Multiforme (GBM)
Head and Neck Squamous Cell Carcinoma (HNSC)
Kidney Chromophobe Renal Cell Carcinoma (KICH)
Kidney Renal Clear Cell Carcinoma (KIRC)
Kidney Renal Papillary Cell Carcinoma (KIRP)
Lung Adenocarcinoma (LUAD)
Ovarian Serous Cystadenocarcinoma (OV)
Prostate Adenocarcinoma (PRAD)
Rectum Adenocarcinoma (READ)
Skin Cutaneous Melanoma (SKCM)
Stomach Adenocarcinoma (STAD)
Thyroid Carcinoma (THCA)
Uterine Corpus Endometrioid Carcinoma

**Supplementary Table 2. The 32 genes that comprised the genetic signature**

*ACTA2*
*AREG*
*ASCC2*
*BEST1*
*BRCA1*
*CREBBP*
*DDX5*
*EP300*
*ESR1*
*FAM96A*
*FHL2*
*GNL3*
*HIPK2*
*HSF1*
*IGSF9*
*JUN*
*MSH6*
*NCOA6*
*NCOA6IP*
*PARP1*
*PAWR*
*PCNA*
*PML*
*PPP2R5A*
*RPA1*
*SMAD3*
*SMARCA4*
*TP53*
*TP63*
*WRN*
*WT1*
*WTAP*

**Supplementary Table 3: Variable selection using a regularized Cox model of the Yonsei cohort.** [1]P value for the interaction term is based on the regularized Cox proportional hazards model.

| Characteristics | Hazard Ratio (95% CI) | P value[1] |
|---|---|---|
| **Stage** | | |
| I | Reference | |
| II | 1.00 (0.75, 3.51) | |
| III | 1.82 (1.24, 6.89) | 0.118 |
| IV | 7.89 (3.70, 42.01) | 0.015 |
| **Adjuvant Chemotherapy Use** | | |
| Chemotherapy | Reference | |
| No chemotherapy | 1.00 (0.78, 1.59) | |
| **Age (continuous)** | 1.02 (1.01, 1.04) | 0.002 |
| **Sex** | | |
| Male | Reference | |
| Female | 0.98 (0.72, 1.16) | 0.888 |
| **Lauren Type** | | |
| Intestinal | Reference | |
| Mixed | 0.78 (0.35, 1.29) | 0.553 |
| Diffuse | 1.07 (0.88, 1.52) | 0.761 |
| Other | 1.28 (1.00, 1.95) | 0.238 |
| **Lymphovascular Invasion** | | |
| Negative | Reference | |
| Positive | 1.196 (1.00, 1.61) | 0.245 |
| **Perineural Invasion** | | |
| Negative | Reference | |
| Positive | 1.07 (0.83, 1.54) | 0.731 |
| **Tumor Location** | | |
| Antrum | Reference | |
| Body | 1.00 (0.83, 1.33) | 1.00 |
| Cardia | 1.00 (0.59, 1.38) | 1.00 |
| Whole | 1.32 (0.72, 3.76) | 0.566 |
| **Molecular Subtype** | | |
| Group 1 | Reference | |
| Group 2 | 1.22 (1.00, 2.24) | 0.609 |
| Group 3 | 1.47 (1.00, 2.51) | 0.138 |
| Group 4 | 1.67 (1.01, 2.82) | 0.050 |

**Supplementary Table 4: Multivariate analysis with variables selected by the regularized cox model of the Yonsei cohort.** [1]P value for the interaction term is based on the regularized Cox proportional hazards model.

| Characteristics | Hazard Ratio (95% CI) | P value[1] |
|---|---|---|
| **Age** | | |
| ≤60 years | Reference | |
| ≥60 years | 1.98 (1.54, 2.55) | <0.001 |
| **Stage** | | |
| I | Reference | |
| II | 1.68 (0.60, 4.69) | 0.321 |
| III | 3.17 (1.17, 8.58) | 0.023 |
| IV | 17.57 (5.89, 52.45) | <0.001 |
| **Molecular Subtype** | | |
| Group 1 | Reference | |
| Group 2 | 2.07 (1.39, 3.07) | <0.001 |
| Group 3 | 1.68 (1.10, 2.57) | 0.016 |
| Group 4 | 2.27 (1.53, 3.39) | <0.001 |

**Supplementary Table 5. Comparison of classification systems.** [1]P values were calculated using the Chi-square test.

| Characteristics | Group 1 (Black) | Group 2 (Green) | Group 3 (Blue) | Group 4 (Red) | Total | P value[1] |
|---|---|---|---|---|---|---|
| **Cheong et al, Lancet Oncology, 2018** | | | | | | <0.001 |
| GST | 15 (13.2 %) | 31 (24.0 %) | 21 (13.0 %) | 23 (14.2 %) | 90 (15.9 %) | |
| INF | 27 (23.7 %) | 14 (10.9 %) | 31 (19.1 %) | 22 (13.6 %) | 94 (16.6 %) | |
| INT | 32 (28.1 %) | 33 (25.6 %) | 27 (16.7 %) | 4 (2.5 %) | 96 (16.9 %) | |
| MXD | 30 (26.3 %) | 40 (31.0 %) | 14 (8.6 %) | 14 (8.6 %) | 98 (17.3 %) | |
| MSC | 2 (1.8 %) | 2 (1.6 %) | 32 (19.8 %) | 74 (45.7 %) | 110 (19.4 %) | |
| Missing | 8 (7.0 %) | 9 (7.0 %) | 37 (22.8 %) | 25 (15.4 %) | 79 (13.9 %) | |
| **ACRG** | | | | | | |
| MSS/EMT | 0 (0.0 %) | 6 (8.5 %) | 0 (0.0 %) | 40 (62.5 %) | 46 (15.3 %) | <0.001 |
| MSI | 43 (55.1 %) | 10 (14.1 %) | 12 (13.8 %) | 3 (4.7 %) | 68 (22.7 %) | |
| MSS/TP53+ | 14 (17.9 %) | 23 (32.4 %) | 33 (37.9 %) | 9 (14.1 %) | 79 (26.3 %) | |
| MSS/TP53- | 21 (26.9 %) | 32 (45.1 %) | 42 (48.3 %) | 12 (18.8 %) | 107 (35.7 %) | |

**Supplementary Table 6. Multivariate analysis of Yonsei cohort to include Lancet Oncology subtypes.** [1]P value for the interaction term is based on the Cox proportional hazards model.

| Characteristics | Hazard Ratio (95% CI) | P value[1] |
|---|---|---|
| **Age** | | |
| ≤60 years | Reference | |
| >60 years | 2.01 (1.52, 2.65) | <0.001 |
| **Stage** | | |
| I | Reference | |
| II | 1.44 (0.50, 4.11) | 0.5 |
| III | 3.25 (1.19, 8.89) | 0.022 |
| IV | 16.94 (5.54, 51.76) | <0.001 |
| **Lauren Type** | | |
| Diffuse | Reference | |
| Intestinal | 0.94 (0.66, 1.33) | 0.728 |
| Mixed | 0.71 (0.33, 1.56) | 0.4 |
| Other | 1.32 (0.92, 1.90) | 0.128 |
| **Perineural Invasion** | | |
| Negative | Reference | |
| Positive | 1.15 (0.81, 1.62) | 0.442 |
| **Subtype in Cheong et al, Lancet Oncology, 2018** | | |
| GST | Reference | |
| INF | 0.71 (0.44, 1.14) | 0.158 |
| INT | 0.96 (0.61, 1.52) | 0.857 |
| MXD | 1.47 (0.95, 2.28) | 0.085 |
| MSC | 1.03 (0.66, 1.59) | 0.912 |
| **Molecular Subtype** | | |
| Group 1 | Reference | |
| Group 2 | 1.96 (1.25, 3.07) | 0.003 |
| Group 3 | 1.64 (1.05, 2.57) | 0.03 |
| Group 4 | 2.41 (1.48, 3.94) | <0.001 |

**Supplementary Table 7. Multivariate analysis of risk score in the ACRG cohort.** [1]P value for the interaction term is based on the Cox proportional hazards model.

| Characteristics | Hazard Ratio (95% CI) | P value[1] |
|---|---|---|
| **Age** | | |
| ≤60 years | Reference | |
| >60 years | 1.79 (1.24, 2.57) | 0.002 |
| **Stage** | | |
| I | Reference | |
| II | 1.60 (0.61, 4.17) | 0.340 |
| III | 3.03 (1.18, 7.77) | 0.021 |
| IV | 8.01 (3.13, 20.5) | 0.000 |
| **Tumor Location** | | |
| Antrum | Reference | |
| Body | 0.93 (0.64, 1.35) | 0.688 |
| Cardia | 1.21 (0.74, 2.00) | 0.447 |
| Whole | 1.56 (0.63, 3.86) | 0.335 |
| **Lauren Type** | | |
| Diffuse | Reference | |
| Intestinal | 0.80 (0.56, 1.15) | 0.229 |
| Mixed | 1.51 (0.59, 3.89) | 0.390 |
| **Risk Score (per unit increase)** | 1.08 (1.02, 1.14) | 0.005 |

**Supplementary Table 8. Multivariate analysis of risk score in the Sohn et al cohort.** [1]P value for the interaction term is based on the Cox proportional hazards model.

| Characteristics | Hazard Ratio (95% CI) | P value[1] |
|---|---|---|
| **Age** | | |
| ≤60 years | Reference | |
| >60 years | 1.83 (1.24, 2.70) | 0.002 |
| **Stage** | | |
| I | Reference | |
| II | 1.09 (0.48, 2.47) | 0.832 |
| III | 2.38 (1.29, 4.37) | 0.005 |
| IV | 5.05 (2.68, 9.51) | < 0.001 |
| **Tumor Location** | | |
| Antrum | Reference | |
| Body | 1.12 (0.73, 1.70) | 0.604 |
| Cardia | 0.80 (0.32, 2.01) | 0.630 |
| Fundus | 0.74 (0.29, 1.89) | 0.532 |
| Whole | 4.57 (1.68, 12.5) | 0.003 |
| **Lauren Type** | | |
| Diffuse | Reference | |
| Intestinal | 0.99 (0.65, 1.52) | 0.976 |
| Mixed | 1.32 (0.61, 2.87) | 0.480 |
| **Risk Score (per unit increase)** | 1.10 (1.03, 1.18) | 0.006 |

**Supplementary Table 9. Multivariate analysis of risk score in the TCGA cohort.** [1]P value for the interaction term is based on the Cox proportional hazards model.

| Characteristics | Hazard Ratio (95% CI) | P value[1] |
|---|---|---|
| **Age** | | |
| ≤60 years | Reference | |
| >60 years | 1.91 (1.32, 2.78) | 0.001 |
| **Stage** | | |
| I | Reference | |
| II | 1.59 (0.82, 3.07) | 0.172 |
| III | 2.53 (1.38, 4.66) | 0.003 |
| IV | 4.94 (2.47, 9.88) | 0.000 |
| **Risk Score (per unit increase)** | 1.06 (1.00, 1.11) | 0.046 |

**Supplementary Table 10. Multivariable Cox proportional analysis of age, cancer stage, and chemotherapy treatment in relation to the risk of death in the Yonsei cohort, stratified by consensus clustering subgroup.** [1]P value for the interaction term is based on the Cox proportional hazards model.

| Characteristics | Hazard Ratio (95% CI) | P value[1] |
|---|---|---|
| **Group 1** | | |
| **Age** | | |
| ≤60 years | Reference | |
| >60 years | 4.47 (1.43, 13.93) | 0.01 |
| **Stage** | | |
| I | 1.17 (0.20, 6.93) | 0.864 |
| II | Reference | |
| III | 1.19 (0.45, 3.16) | 0.723 |
| IV | 11.21 (1.43, 88.16) | 0.022 |
| **Lauren Type** | | |
| Diffuse | Reference | |
| Intestinal | 1.26 (0.39, 4.02) | 0.701 |
| Mixed | 0.67 (0.07, 6.58) | 0.733 |
| Other | 1.18 (0.32, 4.29) | 0.806 |
| **Perineural Invasion** | | |
| Negative | Reference | |
| Positive | 0.98 (0.30, 3.23) | 0.973 |
| **Chemotherapy** | | |
| No chemotherapy | Reference | |
| 5-FU alone | 2.25 (0.61, 8.35) | 0.226 |
| 5-FU + platinum | 6.80(1.46, 31.63) | 0.015 |
| 5-FU + others | 2.00 (0.36, 11.08) | 0.429 |
| | | |
| **Group 2** | | |
| **Age** | | |
| ≤60 years | Reference | |
| >60 years | 1.71(0.86, 3.38) | 0.125 |
| **Stage** | | |
| I | 0.00* | 0.997 |
| II | Reference | |
| III | 2.65 (1.12, 6.23) | 0.026 |
| IV | < 0.001 | 0.998 |
| **Lauren Type** | | |
| Diffuse | Reference | |

| | | |
|---|---|---|
| Intestinal | 0.80 (0.37, 1.73) | 0.576 |
| Mixed | 0.25(0.032, 2.00) | 0.192 |
| Other | 1.04 (0.41, 2.64) | 0.928 |
| **Perineural Invasion** | | |
| Negative | Reference | |
| Positive | 0.64 (0.23, 1.73) | 0.375 |
| **Chemotherapy** | | |
| No chemotherapy | Reference | |
| 5-FU alone | 0.37 (0.14, 0.99) | 0.049 |
| 5-FU + platinum | 0.38 (0.09, 1.56) | 0.179 |
| 5-FU + others | 0.69 (0.26, 1.84) | 0.462 |
| | | |
| **Group 3** | | |
| **Age** | | |
| ≤60 years | Reference | |
| >60 years | 1.34 (0.81, 2.22) | 0.257 |
| **Stage** | | |
| I | 2.41 (0.46, 12.59) | 0.296 |
| II | Reference | |
| III | 3.31 (0.63, 17.46) | 0.159 |
| IV | 9.37 (0.78, 113.09) | 0.078 |
| **Lauren Type** | | |
| Diffuse | Reference | |
| Intestinal | 1.31 (0.64, 2.68) | 0.456 |
| Mixed | 0.39 (0.05, 2.99) | 0.366 |
| Other | 1.66 (0.85, 3.23) | 0.135 |
| **Perineural Invasion** | | |
| Negative | Reference | |
| Positive | 2.56 (1.05, 6.25) | 0.040 |
| **Chemotherapy** | | |
| No chemotherapy | Reference | |
| 5-FU alone | 0.99 (0.41, 2.38) | 0.979 |
| 5-FU + platinum | 0.28 (0.08, 0.96) | 0.043 |
| 5-FU + others | 1.28 (0.50, 3.24) | 0.608 |
| | | |
| **Group 4** | | |
| **Age** | | |
| ≤60 years | | |
| >60 years | 1.94 (1.12, 3.38) | 0.018 |
| **Stage** | | |

| | | |
|---|---|---|
| I | 0.00* | 0.997 |
| II | Reference | |
| III | 1.83 (0.89, 3.76) | 0.098 |
| IV | 13.10 (1.47, 117.12) | 0.021 |
| **Lauren Type** | | |
| Diffuse | Reference | |
| Intestinal | 0.91 (0.48, 1.73) | 0.776 |
| Mixed | 1.39 (0.46, 4.19) | 0.564 |
| Other | 1.76 (0.80, 3.86) | 0.161 |
| **Perineural Invasion** | | |
| Negative | Reference | |
| Positive | 1.30 (0.71, 2.37) | 0.391 |
| **Chemotherapy** | | |
| No chemotherapy | Reference | |
| 5-FU alone | 0.73 (0.30, 1.79) | 0.491 |
| 5-FU + platinum | 0.94 (0.37, 2.41) | 0.893 |
| 5-FU + others | 0.85 (0.36, 2.02) | 0.707 |

* very few patients
