# Supplementary material for: Development and validation of a prognostic and predictive 32-gene signature for gastric cancer
Source: Nat Commun. 2022 Feb 9;13:774. doi: 10.1038/s41467-022-28437-y (PMC8828873; doi:10.1038/s41467-022-28437-y)
Supplement: Supplementary file 3 — Description of Additional Supplementary Files [file 41467_2022_28437_MOESM3_ESM.docx]

Description of Additional Supplementary Files

File Name: Supplementary Data 1

Description: Detailed clinical and pathologic variables of 567 patients in the Yonsei cohort

File Name: Supplementary Data 2

Description: Gene set enrichment analysis.

File Name: Supplementary Data 3

Description: Risk score for the ACRG cohort

File Name: Supplementary Data 4

Description: Risk score for the Sohn et al cohort

File Name: Supplementary Data 5
Description: Risk score for TCGA cohort
