# Supplementary material for: Development and validation of a prognostic and predictive 32-gene signature for gastric cancer
Source: Nat Commun. 2022 Feb 9;13:774. doi: 10.1038/s41467-022-28437-y (PMC8828873; doi:10.1038/s41467-022-28437-y)
Supplement: Supplementary file 9 — Reporting Summary [file 41467_2022_28437_MOESM9_ESM.pdf]

nature research

Corresponding author(s): Jae-Ho Cheong, Tae Hyun Hwang

Last updated by author(s): Dec 16, 2021

Reporting Summary

Nature Research wishes to improve the reproducibility of the work that we publish. This form provides structure for consistency and transparency in reporting. For further information on Nature Research policies, see our Editorial Policies and the Editorial Policy Checklist.

Statistics

For all statistical analyses, confirm that the following items are present in the figure legend, table legend, main text, or Methods section.

n/a	Confirmed	
☐	☒	The exact sample size (n) for each experimental group/condition, given as a discrete number and unit of measurement
☐	☒	A statement on whether measurements were taken from distinct samples or whether the same sample was measured repeatedly
☐	☒	The statistical test(s) used AND whether they are one- or two-sided<br>*Only common tests should be described solely by name; describe more complex techniques in the Methods section.*
☐	☒	A description of all covariates tested
☐	☒	A description of any assumptions or corrections, such as tests of normality and adjustment for multiple comparisons
☐	☒	A full description of the statistical parameters including central tendency (e.g. means) or other basic estimates (e.g. regression coefficient) AND variation (e.g. standard deviation) or associated estimates of uncertainty (e.g. confidence intervals)
☐	☒	For null hypothesis testing, the test statistic (e.g. F, t, r) with confidence intervals, effect sizes, degrees of freedom and P value noted<br>*Give P values as exact values whenever suitable.*
☒	☐	For Bayesian analysis, information on the choice of priors and Markov chain Monte Carlo settings
☒	☐	For hierarchical and complex designs, identification of the appropriate level for tests and full reporting of outcomes
☒	☐	Estimates of effect sizes (e.g. Cohen's d, Pearson's r), indicating how they were calculated

Our web collection on statistics for biologists contains articles on many of the points above.

Software and code

Policy information about availability of computer code

Data collection	Data was downloaded from publicly available databases.
Data analysis	The bioinformatics tool NTriPath uses somatic mutation profiles, pathway databases, and gene-gene interaction networks as input and identifies cancer-type-specific associated pathways. NTriPath source code with input datasets are available at the following link: https://github.com/hwanglab/NTriPath. Support vector machine using linear kernel was utilized to build a predictive model to generate risk scores to predict overall survival following surgical resection of gastric cancer. Source code and datasets for risk score prediction is available at the following link: https://github.com/hwanglab/NTriPath.<br><br>The MATLAB and R scripts used for this study are available here: https://github.com/hwanglab/Yonsei_gastric_cancer_32genes. The DOI for the code is 10.5281/zenodo.5779446 [https://zenodo.org/record/5779446#.YbtuSH3ML0o].

For manuscripts utilizing custom algorithms or software that are central to the research but not yet described in published literature, software must be made available to editors and reviewers. We strongly encourage code deposition in a community repository (e.g. GitHub). See the Nature Research guidelines for submitting code & software for further information.

Data

Policy information about availability of data

All manuscripts must include a data availability statement. This statement should provide the following information, where applicable:

- Accession codes, unique identifiers, or web links for publicly available datasets
- A list of figures that have associated raw data
- A description of any restrictions on data availability

Gene expression profiles of patients treated at Yonesi University can be found here: [https://www.ncbi.nlm.nih.gov/geo/query/acc.cgi?acc=GSE183136] and [https://www.ncbi.nlm.nih.gov/geo/query/acc.cgi?acc=GSE84437]. RNA-sequencing data for patients treated with immune checkpoint inhibitors is available herein the European Genome-Phenome Archive under the Dataset ID EGAD00001008091: [https://ega-archive.org/studies/EGAS00001005588]. The ACRG data file is available here: [https://www.ncbi.nlm.nih.gov/geo/query/acc.cgi?acc=GSE62254]. Data from the Sohn et al cohort is available here: [https://www.ncbi.nlm.nih.gov/geo/query/acc.cgi] and [https://www.ncbi.nlm.nih.gov/geo/query/acc.cgi]. Data from the Kim et al cohort is available here: [https://www.ebi.ac.uk/ena/browser/view/PRJEB25780].

Field-specific reporting

Please select the one below that is the best fit for your research. If you are not sure, read the appropriate sections before making your selection.

☒ Life sciences ☐ Behavioural & social sciences ☐ Ecological, evolutionary & environmental sciences

For a reference copy of the document with all sections, see nature.com/documents/nr-reporting-summary-flat.pdf

Life sciences study design

All studies must disclose on these points even when the disclosure is negative.

Sample size	This was a retrospective study and no sample size calculation was performed. All gastric adenocarcinoma patients who underwent surgical resection at Yonsei University from 1999-2020 were included in the study. Additionally, 45 patients treated with immunotherapy at Seoul St. Mary's (2018-2020) Hospital and Yonsei University (2014-2017) were included in the study.
Data exclusions	No data was excluded from the analysis.
Replication	The molecular classification system and risk score that were developed was validated in 3 independent cohorts.
Randomization	Patients were allocated into groups by molecular subtype using the 32-gene signature that we developed. All covariates are accounted for in univariate and multivariable Cox proportional-hazard analysis.
Blinding	Blinding was not relevant to the study as it was retrospective in nature.

Reporting for specific materials, systems and methods

We require information from authors about some types of materials, experimental systems and methods used in many studies. Here, indicate whether each material, system or method listed is relevant to your study. If you are not sure if a list item applies to your research, read the appropriate section before selecting a response.

Materials & experimental systems

n/a	Involved in the study	
☒	☐	Antibodies
☒	☐	Eukaryotic cell lines
☒	☐	Palaeontology and archaeology
☒	☐	Animals and other organisms
☐	☒	Human research participants
☒	☐	Clinical data
☒	☐	Dual use research of concern

Methods

n/a	Involved in the study	
☒	☐	ChIP-seq
☒	☐	Flow cytometry
☒	☐	MRI-based neuroimaging

Human research participants

Policy information about studies involving human research participants

Population characteristics	567 gastric adenocarcinoma patients who underwent resection at Yonsei University. Detailed information can be found in Table 1. 51.5% of the population was >60 years of age and 68.1% of patients were male. 3.7% of patients had stage I disease, 25.9 stage II, 66.8 stage III, and 3.5% stage IV. Additionally, 45 patients treated with immunotherapy at Seoul St. Mary's (2018-2020) Hospital and Yonsei University (2014-2017) were included in the study.

Recruitment	Retrospective study of 567 gastric adenocarcinoma patients who underwent surgical resection at Yonsei university from 1999-2010 and 45 patients treated with immunotherapy at Yonsei University and Seoul St. Mary's Hospital between 2018-2020.
Ethics oversight	College of Medicine at the Catholic University of Korea Institutional Review Board, Yonsei University Institutional Review Board

Note that full information on the approval of the study protocol must also be provided in the manuscript.
